# Spontaneous Subdural Hematoma Associated with Kasabach-Merritt Syndrome: A Case Report

**DOI:** 10.5505/tjh.2012.78736

**Published:** 2012-10-05

**Authors:** Ufuk Emre, Ayla Gökmen, Banu Özen, Enes Demiryürek, Şanser Gül, Dilvin Gökçe

**Affiliations:** 1 Bülent Ecevit University, School of Medicine, Department of Neurology, Zonguldak, Turkey; 2 Bülent Ecevit University, School of Medicine, Department of Hematology, Zonguldak, Turkey; 3 Bülent Ecevit University, School of Medicine, Department of Neurosurgery, Zonguldak, Turkey; 4 Zonguldak Ataturk State Hospital, Zonguldak, Turkey

## TO THE EDITOR

Kasabach-Merritt syndrome (KMS) is characterized by consumption coagulopathy (thrombocytopenia, anemia, hypofibrinogenemia, and high D-dimer level), angioma, and kaposiform hemangioendothelioma [1,2,3]. Herein we present the first reported case of spontaneous subdural hematoma associated with KMS. Written informed consent was obtained from the patient. 

A 24-year-old female with KMS was referred to our hospital with a subdural hematoma. Persistent, severe headache and neck pain began 10 days earlier, and she had a negative history of trauma. She had migraine without aura, psoriasis, and KMS. KMS was diagnosed at another medical center. Her general physical examination showed large cutaneous hemangiomas in her extremities, with left side predominance. Two fingers on her left hand had been amputated. Her neurological examination was normal. Blood analysis findings were thrombocytopenia (platelet count: 87x10^9^/L), hypofibrinogenemia, and a high D-dimer level (5000 ng/ mL [normal: 147 ng /mL]). The fibrinogen level was 40 mg /dL (normal range: 175-400 mg/ dL). Other hemogram parameters (hemoglobin level: 12.6 g/dl, white blood cell count: 6.1x10^9^/L, hematocrit level: 36.1 %) and laboratory findings were normal. Cranial MRI showed a bilateral subacute subdural hematoma in the frontotemporoparietal region and subacute hemorrhage in the right tentorial region (Figure A-C). Fresh frozen plasma (3 units) was administered to treat hypofibrinogenemia within 3 days after admission, and the patient was given bed rest with head elevated for 15 days.Amitriptyline and analgesic drugs were initiated from the first day of admission. Two months later after discharge from hospital, she did not state any complaints and cranial CT showed a reduction in the size of the subdural hematoma. 

Central nervous system involvement in KMS is rare. A search of the literature showed that there are no reports of intracranial bleeding as a complication of KMS. In the presented case cranial MRI did not show a hemangioma or tumoral lesion in the parenchymal region, but did show a large bilateral subdural hematoma. In patients with a subdural hematoma imaging findings (hematoma volume, degree of midline shift, and compression of the brainstem) and neurological examination (low Glasgow Coma Scale score) may help indicate prognosis and appropriate treatment options (surgery or medical) [4]. In the presented case surgical treatment wasn’t considered because hematoma size didn’t increase and patient’s clinic did not deteriorate during follow-up. Corticosteroid is considered the first-line therapy for KMS—either high-dose methylprednisolone (30 mg/kg for 3 d, followed by dose tapering) or oral corticosteroid (2–5 mg/kg per day), and intravenous corticosteroid may be more efficacious than oral corticosteroid [5]. Alfa-IFN-2a and chemotherapeutic agents are used to prevent tumor expansion [1,5]. Packed red cells, fresh frozen plasma, cryoprecipitate, and pentoxifyline may be considered for treatment [6]. Fresh frozen plasma (15 mL/kg) is recommended in patients with prolonged clotting time and generalized bleeding, and prior to invasive procedures [2]. Surgical excision, embolization, and radiotherapy can be used in appropriate patients [1,2]. The presented patient received medical therapy based on the clinical findings. In conclusion, the present case report is the first to describe a subdural hematoma in a KMS patient; thus, KMS should be considered in the differential diagnosis of patients with intracranial hemorrhage. 

## CONFLICT OF INTEREST STATEMENT

None of the authors has any conflicts of interest, including specific financial interests, relationships, and/or affiliations, relevant to the subject matter or materials included.

## Figures and Tables

**Figure 1:**
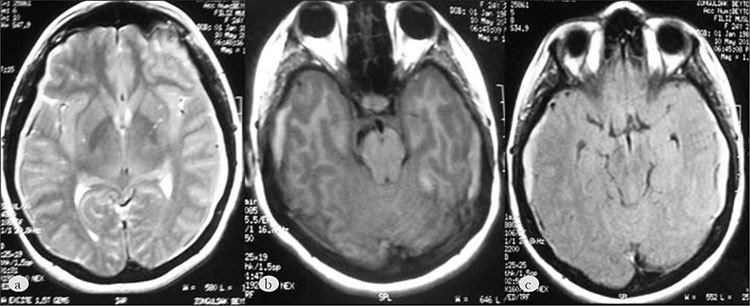
The cranial magnetic resonance imaging revealed bilateral subacute subdural haematomas in the frontotemporoparietalregion on axial T2-weighted image (a), T1-weighted (b), FLAIR image (c) respectively.
